# A Novel Framed Slotted Aloha Medium Access Control Protocol Based on Capture Effect in Vehicular Ad Hoc Networks

**DOI:** 10.3390/s24030992

**Published:** 2024-02-03

**Authors:** Lianyou Lai, Zhongzhe Song, Weijian Xu

**Affiliations:** School of Ocean Information Engineering, Jimei University, Xiamen 361000, China; kaikaixinxinlly@jmu.edu.cn (L.L.); 202111810010@jmu.edu.cn (Z.S.)

**Keywords:** FSA, slotted Aloha, capture effect, Rician, vehicular ad hoc networks (VANETs), medium access control (MAC)

## Abstract

The capture effect is a frequently observed phenomenon in vehicular ad hoc networks (VANETs) communication. When conflicts arise during time slot access, failure to access does not necessarily occur; instead, successful access may still be achieved. The capture effect can enhance the likelihood of multiple access and improve communication efficiency. The security of VANETs communication is undoubtedly the primary concern. One crucial approach to enhance security involves the design of an efficient and reliable medium access control (MAC) protocol. Taking into account both aspects, we propose a novel framed slotted Aloha (FSA) MAC protocol model. Firstly, we derive the closed-form expression for the capture probability in the Rician fading channel in this paper. Subsequently, we analyze how the number of vehicles and time slots influence the success probability of vehicle access channels as well as examine the impact of the capture effect on this success probability. Then, under constraints regarding vehicle access channel success probability, we derive optimal values for slot numbers, access times, and transmission power while proposing a comprehensive implementation method to ensure high access channel success probabilities. We verify both theoretical derivations and proposed methods through simulation experiments.

## 1. Introduction

With the rapid development of information and communication technology, VANETs have been widely applied in the field of intelligent transportation. The vehicle network protocol is also designed with a layered approach, and the MAC protocol of the vehicle network protocol is particularly important due to security considerations. The Aloha protocol, a multiple-access random access algorithm, plays a crucial role in wireless communication networks [1]. It encompasses the pure-Aloha protocol, slotted Aloha (SA) protocol, and frame slotted Aloha (FSA) protocol [2,3,4]. The FSA is further categorized into basic frame slotted Aloha (BFSA) with fixed frame length and dynamic frame slotted Aloha (DFSA) with variable frame length [5,6].

The performance of various FSA protocols is significantly affected by the capture effect, which arises from the adoption of an access slot contention mechanism [7]. In the FSA protocol, a frame consists of *N* slots, and each user randomly selects one slot within the frame for transmitting data packets. Slot selection follows a method of random selection and free competition. If only one user selects a slot, the access is successful. However, if two or more users select the same slot simultaneously, a collision conflict occurs. In case of collision, access may either fail or succeed depending on the power levels involved. When competing users have similar power levels, access fails; however, when one user’s power level significantly exceeds that of the others in competition, it successfully accesses while others fail to do so. This phenomenon is known as the capture effect [8,9]. The prudent utilization of the capture effect can thus augment the probability of user access.

In recent years, the research on FSA protocol has garnered significant attention [10,11]. The utilization of FSA has been widespread in various domains including wireless sensor networks [12], vehicular ad hoc networks [13,14], and radio frequency identification networks [15]. Meanwhile, an increasing number of end-users are adopting the FSA protocol for communication, leading to intensified competition for access time slots. The phenomenon of capture within the FSA protocol has emerged as a pivotal consideration.

In this paper, the capture probability of the Rician fading channel in FSA-based networks is analyzed, and a novel FSA MAC protocol model based on the capture effect is proposed. Additionally, the access probability is considered as an essential constraint. The key contributions of this study are outlined below.

- The closed-form expression for the acquisition probability is derived in the context of a Rician fading channel.
- The paper presents an FSA MAC protocol model based on the capture effect and conducts an analysis of the impact of vehicle quantity and slot allocation on the success rate of vehicle access channels.
- The impact of the capture effect on the success rate of the vehicle access channel is analyzed, and the numerical results validate the accuracy of the theoretical analysis.
- The relationship between the number of vehicles and time slot allocation under maximum conflict probability is derived, providing a foundation for the rational distribution of time slots.
- Under the constraint of a vehicle access channel success probability, this paper derives the requirements for slot number, access times, and transmission power. Taking into account comprehensive factors, an implementation method is proposed to comprehensively enhance the success rate of vehicle access channels from these three aspects.

The remaining sections of this article are organized as follows: Section 2 presents an overview of the research work related to this paper. In Section 3, we derive a closed-form expression for the capture probability in a Rician fading channel. Section 4 proposes an FSA MAC protocol model based on the capture effect and analyzes the impact of the number of vehicles and slots on the success rate of vehicle access channels. We present numerical results in Section 5. Finally, we summarize this article in Section 6.

## 2. Related Work

The medium access control (MAC) protocol plays a pivotal role in wireless networks, exerting a direct impact on their performance. Numerous approaches have been proposed from diverse perspectives.

The paper proposed an FSA protocol with variable frame length in ref. [4], taking into account the channel capture effect. By deriving the expression of the optimal frame length from maximum channel utilization, this study focuses on effectively utilizing the channel. In [16], a novel approach is presented for estimating the population of active nodes using an additive scheme, and this estimate is utilized to optimize throughput by determining frame size and participation probability. In [7], a comprehensive analysis of the correlation between the normalized offered load and the attainable performance is provided in terms of packet loss rate. In [17], the authors focus on the case of messaging from mobile vessels to stationary control centers on land through advanced channel random access schemes that exploit time diversity. This paper extends an existing theoretical framework to evaluate the probability of packet loss in FSA systems by examining both the probability distribution of colliding users within a frame and their replicas in time slots. In [18], the authors investigate the congestion behavior of a IEEE802.11p/1609.4-based MAC protocol by varying the vehicle density under urban and highway conditions. In ref. [19], an adaptive and on-demand TDMA-based MAC protocol is proposed to address the challenges of high-transmission collisions and channel resource wastage in the unbalanced traffic conditions of VANETs. In ref. [20], an analytical model based on the Markov chain model is presented to evaluate the performance of IEEE 802.11 MAC for VANETs in the presence of the capture effect. The study investigates various parameters that can potentially impact the performance and establishes their relationship with performance metrics, including the probability of successful transmission, probability of frame capture, and throughput expressions. In ref. [21], the proposed novel scheme can be seamlessly integrated with IEEE 1609.4 to operate on top of IEEE 802.11p infrastructure. The core concept in the proposed solution revolves around distributing access time initiation across the control channel (CCH) period. This technique, an enhanced iteration of the S-Aloha protocol, effectively mitigates collision risks. The MAC protocol based on framed slotted Aloha for these networks is proposed in ref. [22]. The paper investigates the probable packet sizes, energy consumption, battery lifetime, and success rate of our protocol. In ref. [23], the proposed Coordinated multichannel MAC (C-MAC) protocol incorporates the contention-free broadcasting of safety messages. In our C-MAC, vehicles utilize dynamic framed slotted ALOHA (DFSA) to transmit their channel reservation requests during dedicated channel reservation slots. The paper proposes a low-energy low-latency MAC (LL-MAC) protocol, referred to as the low-energy consumption and low-delay MAC protocol in ref. [24], which is based on receiver-initiated and captured effects. This protocol enables rapid matching between the senders and receivers when the sending nodes have data to transmit. Additionally, an enhanced greedy algorithm is introduced for power allocation among nodes, while an efficient collision response mechanism is employed for effective data transmission. In ref. [25], an MAC algorithm is proposed that utilizes the average waiting time as a common control reference, facilitating nodes to achieve equitable channel access by modifying one of the parameters of the IEEE 802.11-enhanced distributed channel access: contention window, arbitration inter-frame space, or transmission opportunity.

In the aforementioned literature, none of them considered the success rate of access probability as a significant security constraint. The closed-form expression for the acquisition probability in the Rician fading channel is derived in this paper. We establish an FSA system model where *N* vehicles compete freely for *L* slots. We analyze the impact of the number of vehicles and slots, as well as the capture effect, on the success probability of vehicle access. From a security perspective, we propose that meeting the constraint condition of successful vehicle access to roadside units is essential. Under this condition, we derive requirements for the number of slots, access times, and vehicle transmission power.

## 3. Capture Probability of Rician Fading Channel

The PDF of the received signal power of Rice channel fading is

(1) $$f_{p}\left(p\right)=\frac{1}{\sigma^{2}}e^{-\frac{p}{\sigma^{2}}}e^{-K}I_{0}\left(\sqrt{\frac{4Kp}{\sigma^{2}}}\right)$$

where *p* represents the instantaneous power of the received signal, $\sigma^{2}$ represents the power of the received signal’s random reflection component, and *K* is the Rician factor. As shown in Equation (1), the power of the Rician fading channel follows a non-centrosymmetric Chi square distribution. When the Rician factor K=0 corresponds to a Rayleigh fading channel.

Under normal circumstances, when multiple signals are simultaneously connected to a channel, a collision effect occurs, resulting in the failure of collectively accessing these signals. However, if the power of one dominant signal is significantly higher—meaning that the ratio between its power and the sum of all other signals’ powers exceeds a certain threshold—and all other signals can be considered as negligible noise, then the successful connection of the dominant signal can still be achieved. This phenomenon is known as the capture effect.

Consider a scenario where *n* signals are simultaneously connected to a channel, and one of these signals is designated as the main signal with the maximum power value denoted by $p_{t}$. When the ratio of $p_{t}$ to the sum of powers from the remaining n−1 signals exceeds a threshold value of *z*, it is considered significantly larger. In such cases, an acquisition effect occurs, resulting in successful access for $p_{t}$ while causing access failure for the other n−1 signals. The mathematical representation is given by Equation (2).

(2) $$\frac{p_{t}}{\sum_{j=1}^{n-1}p_{j}}>z.$$

The probability of capture effect is shown in Equation (3)

(3) Pcapn=n∫0∞fptpt·Prptpt∑j=1n−1pj∑j=1n−1pj>zdpt=n∫0∞fptpt·∫0ptptzzfpn−1pn−1dpn−1dpt.

According to the reference [26], the probability density function (PDF) of the capture effect in a Rician fading channel is presented in Equation (4), while the probability of capture in the Rice fading channel is given by Equation (5).

(4) fpnpn=1+Kp¯nn+1n+122e−nKpnnKn−1n−122exp−pn1+Kp¯n·In−121+KnKpnp¯n

(5) $$\begin{array}{c} p_{cap}\left(n,z,K\right)=n\left\{\begin{array}{c} 1-e^{-nK}\cdot \sum_{i=0}^{\infty }\frac{{\left[\left(n-1\right)K\right]}^{i}}{i!}\cdot \sum_{k=0}^{n-2+i}\frac{{\left(\frac{1}{z+1}\right)}^{k}}{k!}\cdot \\ \;\sum_{j=0}^{\infty }\frac{K^{j}\left(k+j\right)!}{{\left(j!\right)}^{2}}\cdot {\left(\frac{z}{z+1}\right)}^{j+1} \end{array}\right\} \end{array}$$

The function $p_{cap}$ in the Rice fading channel is dependent on the variables *n*, *z*, and *K*. In the case where the Rice factor k=0, the Rice fading channel transforms into a Rayleigh fading channel.

## 4. System Model and Access Probability

### 4.1. Access Model Description

**Figure 1:**
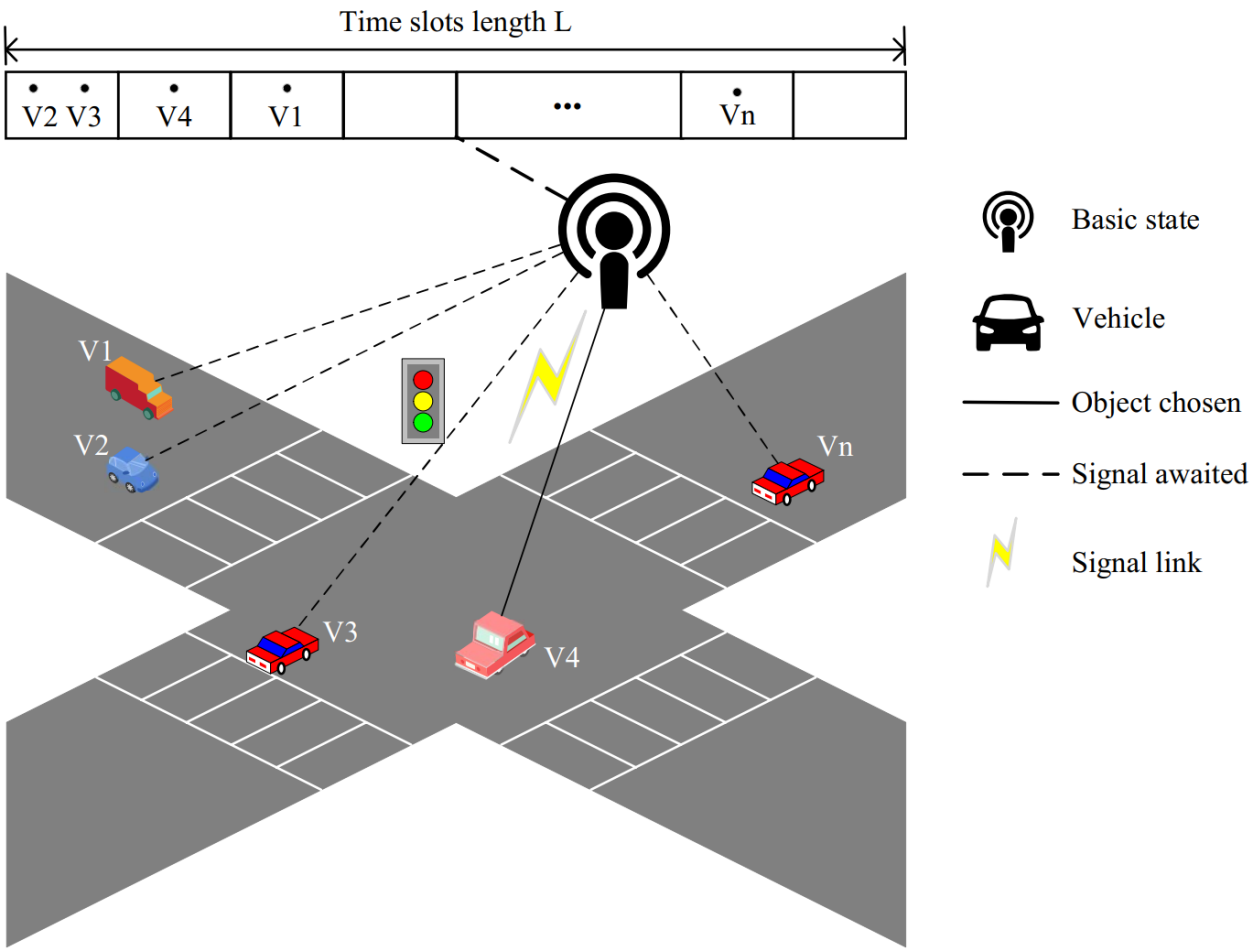
System model (*n* vehicles compete for *L* slots).

The system model depicting the vehicle’s access to the roadside unit is illustrated in Figure 1. A total of *N* vehicles engage in a competitive allocation of *L* time slots among the available roadside units. Each individual vehicle has the flexibility to select any *L* idle time slots, with an equal probability 11LL assigned to each selection.

In Figure 1, *L* represents the count of available idle time slots for access, and *N* denotes the total number of vehicles. The regulations governing vehicle access to the roadside unit are as follows. If no vehicle selects a time slot to access, the time slot remains idle. In the case where only one vehicle chooses to access a time slot, there is no competition conflict, resulting in the successful occupation of the slot. However, if two or more vehicles attempt to access the same time slot simultaneously, a competition conflict arises which may lead to either successful or failed access. The probability of success or failure depends on the capture probability in this scenario. Notably, as the number of vehicles competing for a particular time slot increases, the capture probability decreases and vice versa.

### 4.2. Access Success and Conflict Probability without Considering Capture Effect

*N* vehicles engage in a competitive selection process for the *L* slots of roadside units, with each vehicle exercising its discretion. The total number of possible combinations is denoted as Equation (6)

(6) $$N_{cmb}=L^{N},L\geq 1,N\geq 1.$$

If only one vehicle accesses a specific slot, there is no competition conflict. The number of successful access combinations is denoted by

(7) $$N_{col}^{0}=\left(\begin{array}{c} N-1\\ 0 \end{array}\right)L{\left(L-1\right)}^{N-1}=L{\left(L-1\right)}^{N-1}$$

where $N_{col}^{0}$ represents the number of combinations without conflicts. In this context, it signifies that a vehicle is not combined with other N−1 vehicles, resulting in $\left(\begin{array}{c} N-1\\ 0 \end{array}\right)$ possible combinations. Subsequently, there are *L* available slots for making a free choice, providing *L* options. The remaining N−1 vehicles have the freedom to choose from the available L−1 slots, resulting in a total of ${\left(L-1\right)}^{N-1}$ possible choices. Thus, there are $\left(\begin{array}{c} N-1\\ 0 \end{array}\right)L{\left(L-1\right)}^{N-1}$ combinations.

In a given time slot, *n* vehicles simultaneously attempt to gain access, resulting in competition conflicts. The number of combinations in such scenarios is calculated as Equation (8)

(8) $$N_{col}^{n}=\left(\begin{array}{c} N-1\\ n-1 \end{array}\right)L{\left(L-1\right)}^{N-n},n\leq N$$

where $N_{col}^{n}$ represents the count of combinations involving conflicts among *n* vehicles, wherein all *n* vehicles attempt to select a slot simultaneously. The meaning of this expression is that a certain vehicle is combined with any n−1 vehicles among the set of N−1 vehicles. The total number of possible combinations is determined by the value of $\left(\begin{array}{c} N-1\\ n-1 \end{array}\right)$. Subsequently, exercise your autonomy by selecting freely among *L* available options. There exist *L* alternatives to choose from. The remaining N−n vehicles have the freedom to choose from the remaining L−1 slots, resulting in a total of ${\left(L-1\right)}^{N-n}$ possible choices. Overall, there are $\left(\begin{array}{c} N-1\\ n-1 \end{array}\right)L{\left(L-1\right)}^{N-n}$ combinations available. In the absence of multiple vehicles vying for a slot, there is no occurrence of competition conflict. The probability of successfully accessing the slot remains

(9) $$p_{acc}=p_{col}^{0}=\frac{N_{col}^{0}}{N_{cmb}}={\left(\frac{L-1}{L}\right)}^{N-1}.$$

When multiple vehicles concurrently attempt to access a slot, the likelihood of encountering competition conflicts is

(10) $$p_{col}^{n}=\frac{N_{col}^{n}}{N_{cmb}}=\frac{\left(\begin{array}{c} N-1\\ n-1 \end{array}\right){\left(L-1\right)}^{N-n}}{L^{N-1}}.$$

A slot is selected by multiple vehicles, and the likelihood of conflicts is

(11) $$\begin{array}{c} p_{col}=p\left(i\geq 2\right)\;=\sum_{i=2}^{N}p_{col}^{i}\;=\sum_{i=2}^{N}\frac{\left(\begin{array}{c} N-1\\ i-1 \end{array}\right){\left(L-1\right)}^{N-i}}{L^{N-1}}. \end{array}$$

According to Equation (9), we can obtain

(12) $$\frac{\partial }{\partial L}\ln p_{acc}=\frac{\partial }{\partial L}\ln{\left(\frac{L-1}{L}\right)}^{\left(N-1\right)}=\frac{N-1}{L\left(L-1\right)}>0.$$

Therefore, $p_{acc}$ monotonically increases with *L*. The access probability $p_{acc}$ also increases as the value of *L* increases.

According to Equation (10), let $\frac{\partial }{\partial L}\ln p_{col}^{n}=0$, then obtain the Equation (13)

(13) $$L=\frac{\left(n-1\right)!\left(N-1\right)}{\left[\left(n-1\right)!-1\right]N-\left[\left(n-1\right)!-n\right]}.$$

The maximum value of $p_{col}^{n}$ occurs at this point in time. It represents the probability that *n* vehicles select the same slot for collision, which is maximized. When $p_{col}^{2}$, $p_{col}^{3}$, and $p_{col}^{4}$ are maximum, the relationship between *L* and *N* is shown in Equations (14)–(16)

(14) $$L=N-1\begin{array}{ccc} & s.t. & \max p_{col}^{2} \end{array}$$

(15) $$L=\frac{2\left(N-1\right)}{N+1}\begin{array}{ccc} & s.t. & \max p_{col}^{3} \end{array}$$

(16) $$L=\frac{6\left(N-1\right)}{5N-2}\begin{array}{ccc} & s.t. & \max p_{col}^{4}. \end{array}$$

### 4.3. Access Success and Failure Probability When Considering Capture Effect

When considering the capture effect, successful access may occur when multiple vehicles simultaneously contend for a slot. The probability of achieving successful access under such circumstances is enhanced due to the capture effect. The conditional probability in cases of competition conflict can be represented by Equation (17).

(17) $$\Delta p_{suc}=p_{cap}p_{col}=\sum_{i=2}^{N}p_{col}\left(i\right)p_{cap}\left(i\right).$$

When considering the capture effect, the probability of successful slot access can be expressed as Equation (18), while the probability of slot access failure is represented by Equation (19).

(18) $$p_{suc}=p_{acc}+\Delta p_{suc}=p_{acc}+\sum_{i=2}^{N}p\left(i\right)p_{cap}\left(i\right)$$

(19) $$p_{fail}=p_{col}-\Delta p_{suc}=p_{col}\left(1-p_{cap}\right)=\sum_{i=2}^{N}p_{col}^{i}\left(1-p_{cap}^{i}\right)$$

### 4.4. Access Methods When Access Probability Constraints

Safety is the paramount concern in VANETs, with numerous factors influencing it. Among these, ensuring the timely connectivity of vehicles to VANETs through roadside units stands out as the most crucial aspect. Access delay and access success rate are two key dimensions of this issue for vehicles. A low delay implies a high access success rate and vice versa. Therefore, enforcing a constraint on the vehicle’s access success rate to roadside units becomes imperative for guaranteeing VANET safety. The threshold value for this success rate may vary based on different road conditions; however, addressing this problem is essential. This paper proposes three approaches to enhance the access success rate.

#### 4.4.1. Increase the Number of Access Slots to Improve the Success Rate of Vehicle Access

Revised sentence: “In this scenario, the probability of multiple vehicles selecting the same time slot simultaneously is reduced, thereby enhancing the success rate of accessing the slot in a single attempt through the comprehensive configuration of time slot resources”.

Assuming that the threshold of the access success rate is $p_{th}$, then

(20) $$p_{suc}=p_{acc}+p_{cap}p_{col}>p_{acc}>p_{th}.$$

According to Equations (9) and (15), Equation (21) can be inferred

(21) $$L>\left\lfloor \frac{1}{1-e^{\frac{\ln\left(p_{th}\right)}{N-1}}}\right\rfloor .$$

where $\left\lfloor \cdot \right\rfloor$ denotes the floor function. When Equation (21) is satisfied, the one-time access success probability of the vehicle can be guaranteed to exceed $p_{th}$.

#### 4.4.2. Improving the Success Rate of Vehicle Access in VANETs by Increasing the Access Times

Theoretically, for a given vehicle V1, the initial attempt to access fails, and the probability of successful access within *n* attempts is described by Equation (22)

(22) $$p_{in}\left(n\right)={\left(1-p_{suc}\right)}^{n-1}p_{suc}.$$

The time interval is Δt. The method of accessing the slot is implemented using an equal time interval cycle mode, without considering the access back time strategy. The time required for the *n*-th successful access is represented as Equation (23)

(23) $$t\left(n\right)=\frac{\left(n-1\right)\Delta t}{p_{in}\left(n\right)}.$$

In the practical application scenario, it is imperative for the vehicle to access the roadside unit slot at a specific time $t\left(n\right)$ as the Equation (24) in order to satisfy the conditional real-time requirements

(24) $$t\left(n\right)<t_{\max}.$$

Therefore, the maximum value of access times, denoted as $n_{\max}$, can be calculated when the access slot interval Δt is constant. In normal circumstances, the maximum duration $t_{\max}$ is determined by vehicle speed and safe distance under road conditions. However, in practice, the TDMA slot cycle time is very short and vehicle travel distance is limited. Thus, it is reasonable for vehicles that fail to access roadside units to attempt multiple accesses in order to improve their success rate.

In the initial scenario, assuming that a total of $N_{1}$ vehicles randomly access $L_{1}$ available slots, the probability of the first vehicle to successfully gain access can be determined using either Equation (9) or (13), denoted by $p_{1}$. The number of vehicles successfully accessing slots after the first random access is $\left\lfloor N_{1}p_{1}\right\rfloor$, and these vehicles occupy the same number of slots. The number of vehicles that have not successfully established a connection can be expressed as $N_{2}=N_{1}-N_{1}p_{1}$, while the remaining slots can be represented by $L_{2}=N_{1}-N_{1}p_{1}$. According to Equation (9) or (13), the probability of the second successful vehicle gaining access is denoted by $p_{2}$. The number of vehicles successfully accessing slots after the second random access is $\left\lfloor N_{2}p_{2}\right\rfloor$, and they occupy $\left\lfloor N_{2}p_{2}\right\rfloor$ time slots. The number of vehicles that have not been successfully accessed is given by $N_{3}=N_{2}-N_{2}p_{2}$, where $N_{2}$ represents the total number of vehicles and $p_{2}$ denotes the probability of successful access. Similarly, the remaining time slots can be calculated as $L_{3}=N_{2}-N_{2}p_{2}$. Furthermore, Equation (9) or (13) provides an expression for the probability ($p_{3}$) of achieving a third successful access to vehicles. The process is $\left(\begin{array}{ccc} N_{1} & L_{1} & p_{1} \end{array}\right)\to \left(\begin{array}{ccc} N_{2} & L_{2} & p_{2} \end{array}\right)\to \left(\begin{array}{ccc} N_{3} & L_{3} & p_{3} \end{array}\right)$.

Equations (25) and (26) present the probabilities of total access failure and total access success, respectively, after three random accesses

(25) $$p_{fail}=\left(1-p_{1}\right)\left(1-p_{2}\right)\left(1-p_{3}\right)$$

(26) $$p_{suc}=1-p_{fail}.$$

#### 4.4.3. Revising the Vehicle Transmission Power to Enhance the Capture Probability and Thereby Improve the Success Rate of Vehicle Access

In accordance with the fundamental principles of the capture effect, augmenting the transmission power of a vehicle can enhance its likelihood of being allocated slots when competing with other vehicles, thereby improving the success rate of vehicle access to slots. When the transmission power $p_{t}$ exceeds $z\sum_{j=1}^{n-1}p_{j}$, it means that the capture effect occurs in the region below $z\sum_{j=1}^{n-1}p_{j}$.

(27) $$\mathrm{Pr}\left[p_{t}<z\sum_{j=1}^{n-1}p_{j}\right]=\mathrm{Pr}\left[p_{t}<z\cdot E\left[\sum_{j=1}^{n-1}p_{j}\right]\right]=\mathrm{Pr}\left[p_{t}<z\cdot \left(n-1\right)E\left[p_{j}\right]\right]$$

where $p_{j}$ obeys a Chi-square distribution with one degree of freedom.

The condition N<L is typically necessary to enhance the access success rate and reduce the access delay, particularly for security information frames. This implies that the likelihood of multiple vehicles contending for the same time slot simultaneously is exceedingly low. Consequently, the number of conflicting vehicles can be limited to a maximum of 4, disregarding scenarios where it exceeds this threshold. When z=2, we can derive

(28) $$\begin{array}{c} \mathrm{Pr}\left[p_{t}<z\sum_{j=1}^{n-1}p_{j}\right]=\mathrm{Pr}\left[p_{t}<z\cdot \left(n-1\right)E\left[p_{j}\right]\right]\;=\mathrm{Pr}\left[p_{t}<6\right]\;=0.9857. \end{array}$$

Equation (28) demonstrates that a 7.8 dB increase in vehicle power guarantees an access success rate exceeding 98%. Moreover, when the vehicle power is amplified by 9 dB, it ensures an access success rate surpassing 99.7%.

The access success probability of a vehicle can be improved by increasing the transmission power, while keeping other conditions unchanged. However, this approach is not an overall optimal solution but rather a priority due to the resulting failure of competitive time slots for other vehicles and decrease in access success rate. Nevertheless, it remains feasible and necessary within the context of security information framework.

#### 4.4.4. Access Method Subject to Constraints on Access Probability

After considering the aforementioned cases comprehensively, it becomes evident that adopting a single method has its limitations. Hence, we propose a comprehensive access approach. The specific methodology is as follows: the time slot length is set to L=2N, with access times generally being three-fold, twice in the high-speed section and four times in the low-speed section. In situations requiring safety information transmission during emergencies, an increase of 7 dB to 9 dB in transmission power is implemented.

## 5. Simulation

In this section, we present the simulation results of the algorithm performance. Most simulations were conducted using both numerical and Monte Carlo methods. The Monte Carlo method was employed to validate the accuracy of the numerical simulation results, with an average value obtained from 10,000 independent random experiments. All simulations were performed using MATLAB 2022b software, with random numbers generated by internal MATLAB function. In the provided simulation figures, the legend for the numerical simulation method is labeled as ‘Ana’, while the legend for the Monte Carlo simulation method is labeled as ‘Sim’.

### 5.1. Simulation of Capture Effect

**Figure 2:**
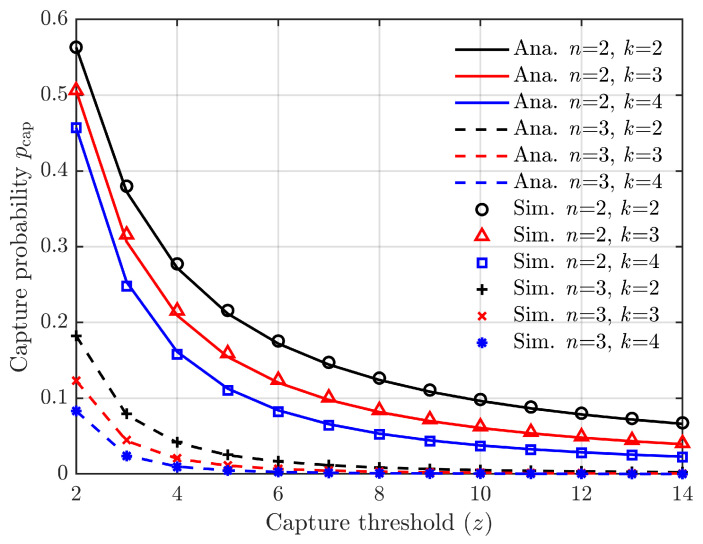
Capture probability vs. power ratio threshold.

The relationship between the acquisition probability and the number of vehicle conflict *n*, the Rician factor *K*, and the power ratio threshold *z* of capture effect is shown in Figure 2 based on Equation (5).

In Figure 2, the relationship between the capture probability $p_{cap}$ and the power ratio threshold *z* is depicted for six combinations of vehicle conflict number *n* and Rician factor *K*. The simulation curves are divided into two groups, with three curves corresponding to n=2 forming one group, and three curves corresponding to n=3 forming the other group. This observation highlights that the impact of *n* on the capture probability $p_{cap}$ outweighs that of *K*. Moreover, when both *K* and *n* remain constant, $p_{cap}$ exhibits a rapid decrease as *z* increases. These findings indicate that $p_{cap}$, particularly when considering values of n≥3, tends to be very small. Additionally, for values of z≥10, $p_{cap}$ also remains negligible. These empirical laws serve as a foundation for approximating the calculation of capture probability.

### 5.2. Simulation of the Model

According to Equations (9) and (10), Table 1 presents the probabilities of successful vehicle access to the roadside unit slot and conflicts arising from 2, 3, or more vehicles simultaneously selecting a slot. The capture effect is not considered in this analysis.

In Table 1, the probabilities of successful access for vehicle 0 under various conditions are presented, including “extreme conditions”, “more vehicles with fewer slots”, “fewer vehicles with more slots”, and “general conditions”, as well as the probability of conflicts arising from multiple vehicles selecting a slot simultaneously. These probabilities can be analyzed quantitatively to assess both successful access and conflict occurrences. Furthermore, they serve as a means to validate the accuracy of the algorithm.

**Figure 3:**
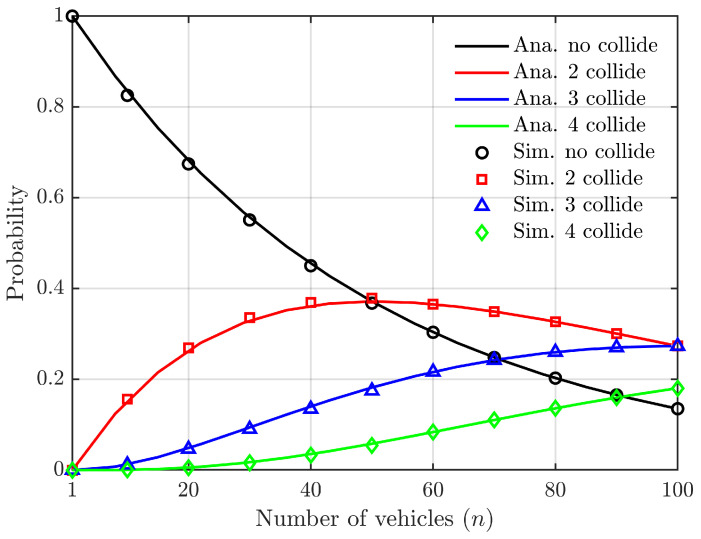
Relationship between access probability, collision probability of *n* vehicles, and the number of vehicles (the slot number is constant, L=50).

According to Equations (9) and (10), Figure 3 depicts the relationship between the probability of successful vehicle access to the roadside unit slot, as well as the probabilities of 2, 3, and 4 vehicles simultaneously selecting a slot leading to conflicts, in relation to the number of vehicles. In this analysis, we do not consider the capture effect and assume a constant time slot length.

In Figure 3, with a fixed number of slots L=50, the range of vehicles *N* varies from 1 to 100. The term “no collide” refers to the scenario where only one vehicle successfully selects and connects to a slot without any conflicts. On the other hand, “2 collide” indicates that two vehicles simultaneously select the same slot, resulting in a conflict. Similar patterns can be observed for other scenarios as well. Figure 3 demonstrates that, when the number of slots remains constant, the probability of successful access without slot selection conflicts decreases as the number of vehicles increases. When *N* is twice as large as *L*, this probability reduces to less than 10%. In cases where there are slot selection conflicts, it is predominantly observed between two or three cars, while conflicts involving four or more cars are relatively rare. Additionally, there is a high likelihood of the capture effect when two or three cars experience slot selection conflicts; however, this effect becomes negligible when four or more cars encounter such conflicts. Therefore, our focus on studying capture effects should primarily revolve around scenarios involving two or three cars slot selection conflicts while disregarding those with four or more cars selections.

**Figure 4:**
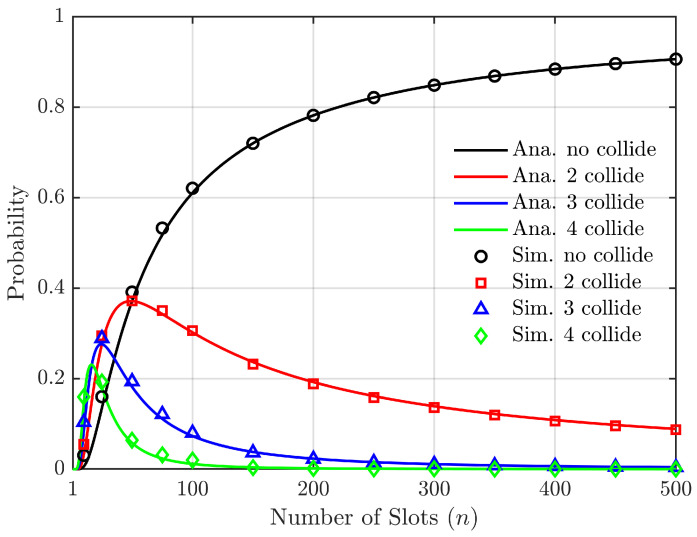
Relationship between access probability, collision probability of *N* vehicles, and slot number (the number of vehicles is constant, N=50).

According to Equations (9) and (10), in the absence of considering the capture effect and with a constant number of vehicles, Figure 4 illustrates the relationship between the probability of successful vehicle access to the roadside unit slot, as well as the probabilities of two, three, and four vehicles simultaneously selecting a slot causing conflicts.

In Figure 4, for a fixed number of vehicles N=50, the range of slots *L* is from 1 to 500. The term “no collide” refers to a scenario where only one vehicle selects a slot and successfully accesses it without any conflict, while “2 collide” indicates that two vehicles select the same slot simultaneously, resulting in a conflict. The remaining cases follow similar patterns. Figure 4 illustrates that, as the number of slots increases, there is a rapid escalation in the probability of slot selection conflicts, with successively increasing probabilities observed for scenarios involving four, three, and two vehicles. Subsequently, this probability gradually decreases but at a slower rate for conflicts between two vehicles. When the number of slots exceeds 100, the likelihood of conflict between car 4 and car 3 becomes negligible and can be disregarded. These findings demonstrate that, when dealing with large numbers of slots and relatively fewer vehicles, the capturing effect primarily considers conflicts between pairs of vehicles while other situations have minimal impact.

**Figure 5:**
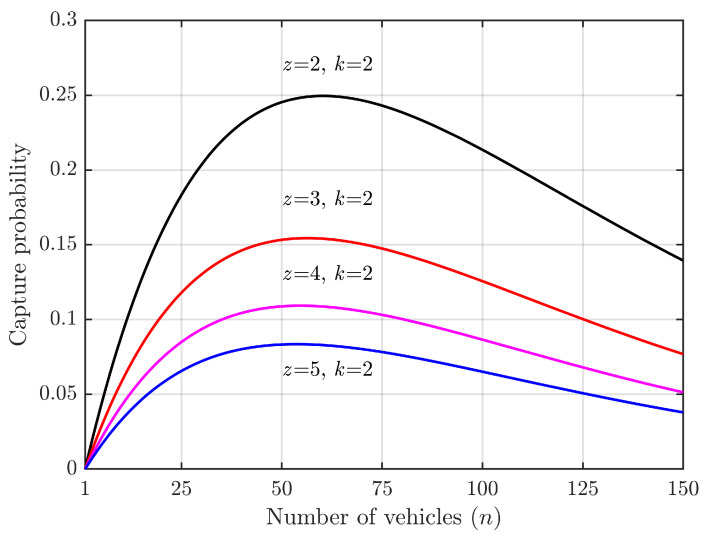
Increment in successful access probabilities due to capture effect vs. number of vehicles.

**Figure 6:**
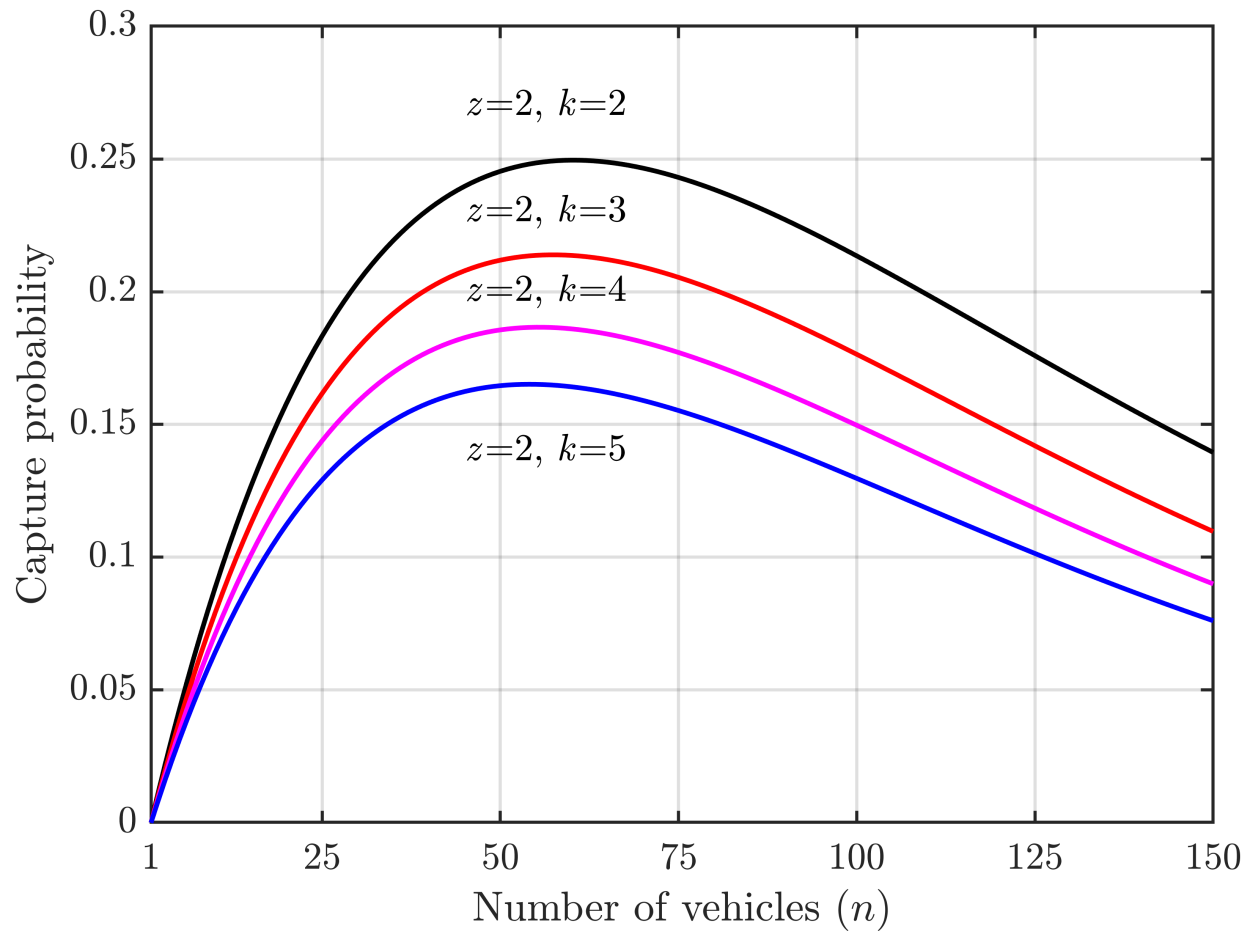
Increment in successful access probabilities due to capture effect vs. number of vehicles.

When considering the capture effect, successful access can occur when multiple vehicles simultaneously contend for a slot. According to Formula (13), the probability of successful access under this condition represents an increment in the probability due to the capture effect. This corresponds to the conditional probability in cases of competition conflict, as illustrated in Figure 5 and Figure 6.

According to Equation (13), the factors affecting the acquisition probability are *L*, *N*, and *K*. In Figure 5 and Figure 6, we set L=50 and varied the number of vehicles, *N*, from 1 to 150. For Figure 5, we fixed K=2 and varied spreading factor, *z*, from 2 to 5. For Figure 6, we fixed z=2 and varied *K* from 2 to 5. Our results show that (1) when there are as many vehicles as slots (N=L), the capture probability is the highest with a significant capture effect; (2) when there are a few vehicles relative to slots (N≪L), acquisition probability is very low due to low collision probabilities; (3) when there are many more vehicles than slots (N≫L), the capture probability decreases due to high collision probabilities but also because competition for access reduces the likelihood of successful captures; (4) comparing Figure 5 and Figure 6, it can be seen that spreading factor has a greater impact on capture probability than does value of *K*.

**Figure 7:**
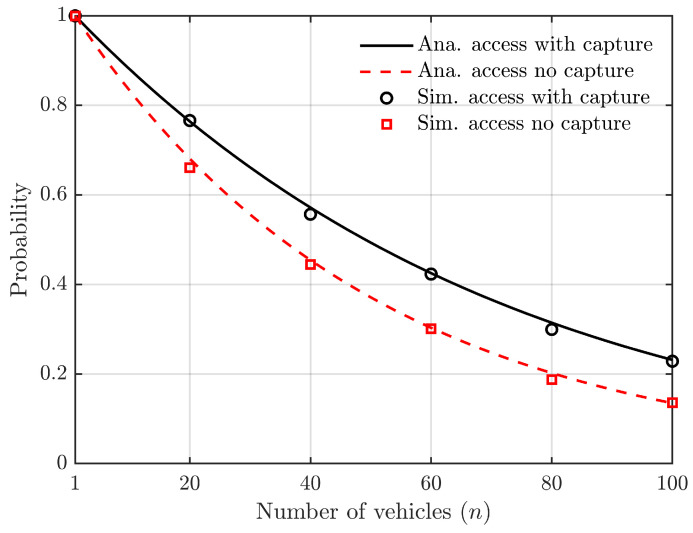
Relationship between the probability of successful access to slots and the number of vehicles (the number of slots is constant).

The relationship between the probability of a vehicle successfully accessing a roadside unit slot and the number of vehicles is depicted in Figure 7, based on Equations (10) and (14).

The simulation parameters in Figure 7 are L=50, z=3, K=3, and the number of vehicles, denoted as *N*, ranges from 1 to 100. As the number of vehicles increases while keeping the number of slots constant, there is an elevated likelihood for multiple vehicles to select the same slot simultaneously, leading to a continuous decline in successful access probability. Figure 7 demonstrates that considering the capture effect significantly enhances the probability of successful vehicle-to-roadside unit access under similar conditions.

**Figure 8:**
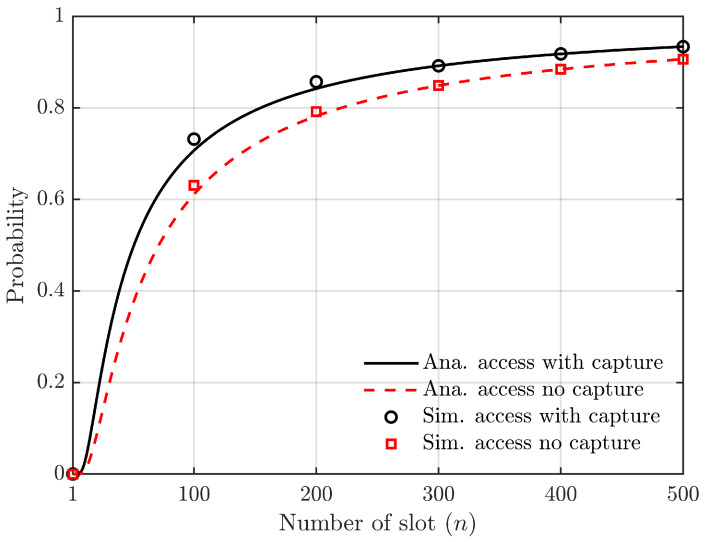
Relationship between the probability of successful access to slots and the number of slots (the number of vehicles is constant).

According to Equations (10) and (14), Figure 8 illustrates the correlation between the probability of successful vehicle access to a roadside unit slot and the number of slots.

The simulation parameters in Figure 8 are N=50, z=3, K=3, and the number of time slots *L* ranges from 1 to 500. As the number of time slots increases while keeping the number of vehicles constant, the likelihood of multiple vehicles selecting the same time slot simultaneously decreases, leading to an increased probability of successful time slot access. Figure 8 demonstrates a significant enhancement in vehicle’s successful access to roadside units when considering the capture effect under similar conditions.

### 5.3. Simulation of Performance

#### 5.3.1. Time Responsiveness

**Figure 9:**
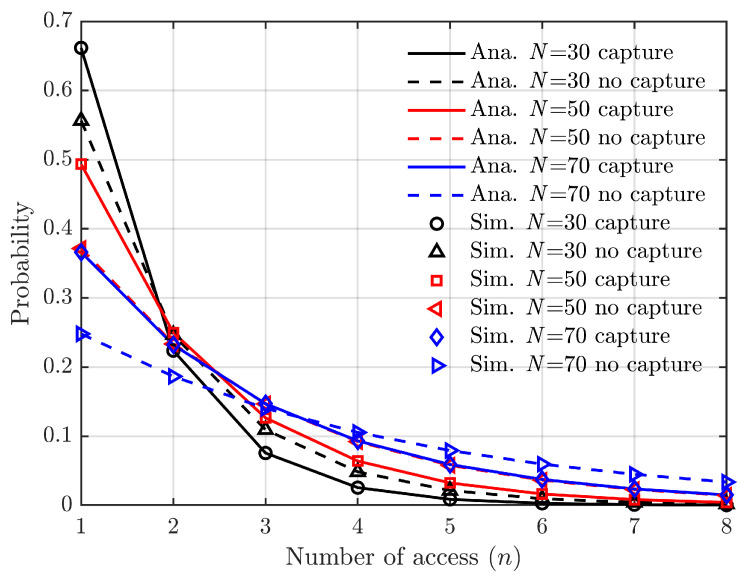
Probability of successful access for the first time.

The probability of successful access for vehicle V1 until the *n*-th attempt is as follows. Figure 9 illustrates a rapid decline in the probability of success for each subsequent access attempt, with the fourth and subsequent attempts having a very low probability of success. Consequently, if an access failure occurs, there is a high likelihood of achieving access success after three consecutive attempts.

**Figure 10:**
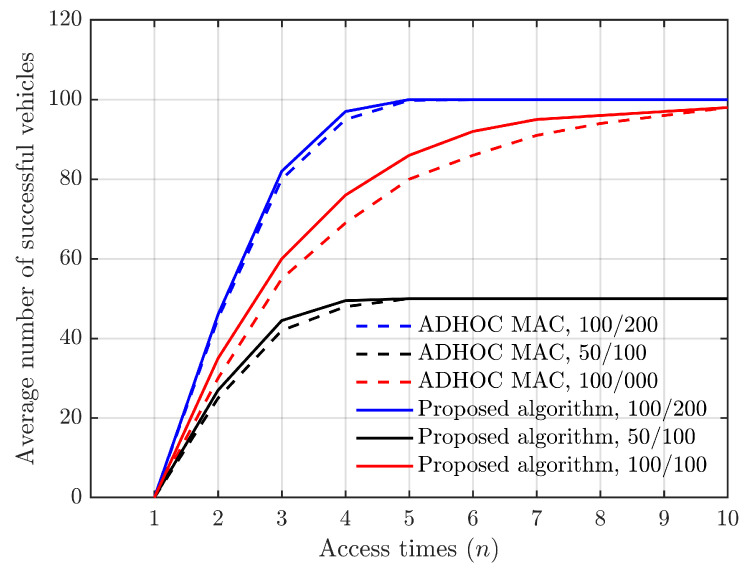
Average number of successful vehicles vs. access time (*n*).

We considered three cases in which the number of vehicles and the number of slots are, respectively, 100/200, 50/100, and 100/100 as Figure 10.

In the 100/200 and 50/100 cases, all the vehicles access successfully within 5 times. In the case 100/100, due to intense competition among vehicles, they need to go through multiple rounds of competition in order to successfully connect with the RSU. The competition for vehicle access time slots is more intense, requiring more attempts to establish connections. Figure 10 meanwhile demonstrates that the proposed method in this paper outperforms ADHOC MAC, particularly when there are many vehicles vying for limited time slots and the competition is fierce. The ADHOC MAC simulation data are sourced from ref. [2].

**Figure 11:**
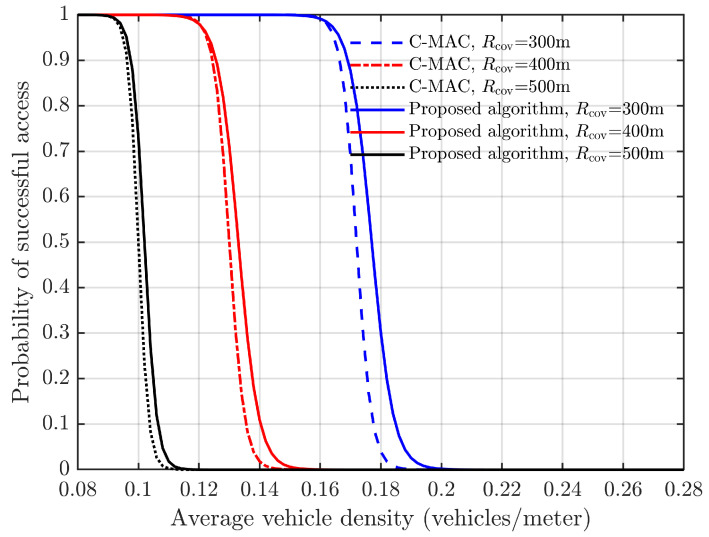
Probability of successful access vs. vehicle density.

We consider a highway equipped with a roadside unit, where vehicles enter and exit the coverage area of the RSU. Assuming a constant speed of 100 km/h for vehicles, the inter-vehicle distance follows a uniform distribution. The RSU coverage ranges are 300 m, 400 m, and 500 m, respectively. Figure 11 illustrates the direct correlation between vehicle density and the successful connection of vehicles to roadside units.

The graph in Figure 11 demonstrates that, at low vehicle densities, the probability of successful access is 1. However, as the vehicle density surpasses a certain threshold, there is a significant decrease in the likelihood of successful access. Specifically, when the RSU coverage range is set to 300 m, the probability of successful access starts declining once the vehicle density exceeds 0.16.

The algorithm demonstrates superiority over the C-MAC algorithm, as depicted in Figure 11. This advantage becomes more pronounced when the RSU coverage range is 300 m. With an increase in vehicle density, the probability of successful vehicle access rapidly decreases. However, compared to the C-MAC method, this decrease occurs at a slower rate due to the capture effect. Consequently, this characteristic renders this algorithm more suitable for urban traffic scenarios characterized by significant fluctuations in vehicle density. The C-MAC simulation data are sourced from ref. [23].

#### 5.3.2. Selection of Parameters under the Constraint of Successfully Accessing Probability

For ensuring security, it is imperative that newly arrived vehicles can access the VANETs in a timely manner through the roadside unit with a high probability of success. In the FSA network, there exist three primary approaches to enhance the vehicle’s access success rate: (1) augmenting the configuration of access slot numbers; (2) mitigating failure probability via multiple accesses; and (3) amplifying the transmission power of vehicles to improve the capture probability.

(1) Increase the number of access slots to improve the success rate of vehicle access.

**Figure 12:**
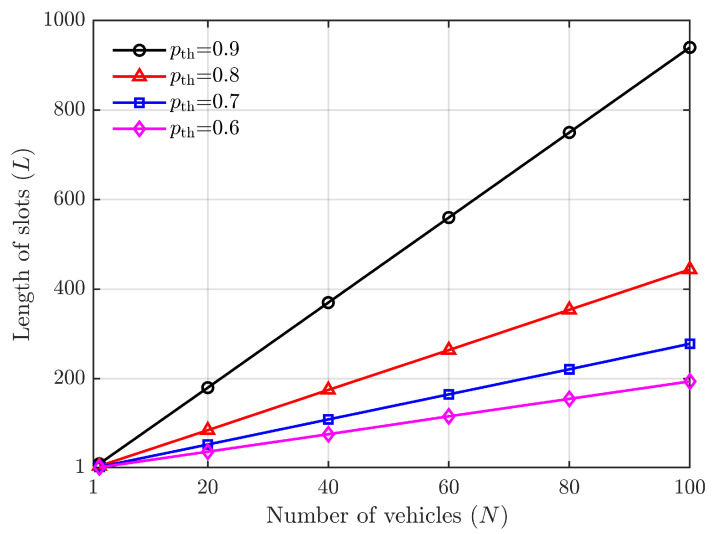
The number of slots vs. the number of the vehicle under the condition of access probability ($p_{th}=0.6,0.7,0.8,0.9$).

The objective of this approach is to prioritize access delay, allocate resources comprehensively, and enhance the success rate of one-time access. The simulation results in Figure 12 demonstrate the impact of different values (0.6, 0.7, 0.8, and 0.9) for $p_{th}$ as per Equation (21).

It can be observed from Figure 12 that a significantly high access success rate can be achieved when the number of slots is abundant. Specifically, with ten times more slots than vehicles, the one-time success rate of vehicle access to the roadside unit exceeds 90%.

(2) Increase access times to improve vehicle access success rate.

The objective of this approach is to comprehensively consider both the delay and the availability of slot resources in order to enhance the access success rate through multiple access. This method requires fewer slot resources, albeit resulting in a higher vehicle access delay to the roadside unit, making it particularly suitable for low-speed scenarios. Based on Equations (25) and (26), the simulation results are presented in Table 2.

The results presented in Table 2 demonstrate that, when the number of slots is twice the number of vehicles, the final access success rate exceeds 98.7% after three consecutive attempts. In comparison to method (1), this approach significantly reduces the number of provided slots while maintaining a remarkably high access success rate.

(3) Enhance the transmission power to elevate the success rate of vehicle access by augmenting the capture probability.

**Figure 13:**
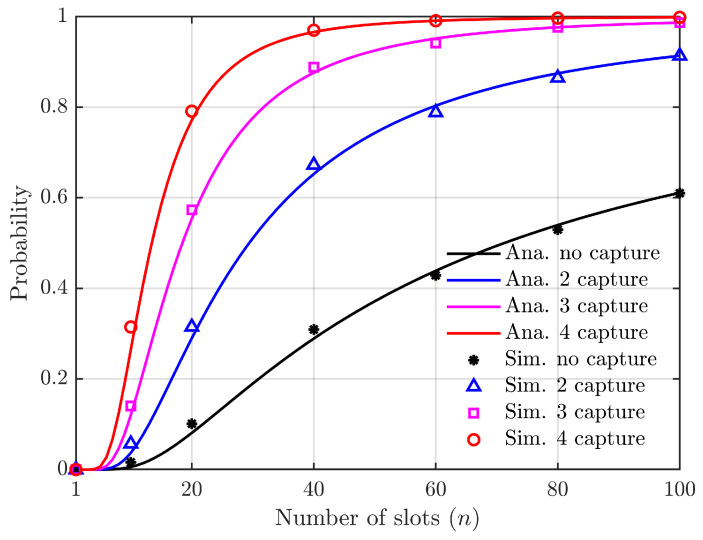
Access success probability vs. the number of slots (*N* = 50).

The capture probability can be enhanced by increasing the transmitting power of the vehicle. Figure 13 illustrates the relationship between the access success probability and the number of slots, considering N=50, as derived from Equations (9), (10) and (27). In this scenario, we account for the capture probability by augmenting the transmission power of vehicles.

The access success probability exhibits a slow increase with the number of slots when the transmission power is small and the capture probability is neglected, as depicted in Figure 13. However, as the transmitting power of vehicles increases, a collision capture effect between two vehicles emerges. Further increasing the transmitting power leads to collision capture effects involving three or four vehicles. Under these circumstances, the access success probability experiences a rapid growth with an increasing number of slots. Remarkably enhanced access success probabilities are observed under identical conditions.

It is worth noting that increasing the transmission power for a specific vehicle can enhance the access success probability. However, this enhancement comes at the expense of reduced success rates for other vehicles’ access attempts. Consequently, while not an overall optimal approach, it becomes indispensable in emergency situations for individual vehicles.

## 6. Conclusions

In this paper, we propose a novel FSA MAC protocol based on the capture effect and derive the closed-form expression of access probability in the Rician fading channel. We establish a system model where *N* vehicles compete for *L* slots, and analyze the impact of vehicle and slot numbers on the success probability of vehicle access. From a security perspective, we suggest that vehicle access to roadside units must adhere to constraints regarding access success rate. Under these conditions, we derive requirements for the slot number, access times, and vehicle transmission power. Finally, through simulation verification, we validate our proposed model and algorithm.

## Figures and Tables

**Table 1:** Illustrative calculations of the probabilities of successful and unsuccessful access.

	*L*	*N*	$p_{\mathrm{col}}^{0}$	$p_{\mathrm{col}}^{2}$	$p_{\mathrm{col}}^{3}$	$p_{\mathrm{col}}^{4}$	$p_{\mathrm{col}}^{5}$
Extreme conditions	1	1	1	0	0	0	0
	1	2	0	1	0	0	0
	1	3	0	0	1	0	0
	2	1	1	0	0	0	0
	2	2	0.50	0.50	0	0	0
	2	3	0.25	0.50	0.25	0	0
More vehicles with fewer slots	10	15	0.22877	0.35586	0.25701	0.11423	0.04413
	10	20	0.13509	0.28518	0.28518	0.17956	0.11500
	10	25	0.07977	0.21271	0.27180	0.22146	0.21426
Fewer vehicles with more slots	30	15	0.62212	0.30033	0.06732	0.009285	0.00095
	30	20	0.52512	0.34404	0.10677	0.020864	0.003201
	30	25	0.44324	0.36682	0.14546	0.036784	0.007688
General conditions	50	40	0.45480	0.36198	0.14036	0.035329	0.007534
	50	50	0.37160	0.37160	0.18201	0.058193	0.016594
	50	60	0.30363	0.36559	0.21637	0.083898	0.030516

**Table 2:** Success probability after three accesses (the number of slots is twice the number of vehicles).

$N_{1}$	$L_{1}$	$p_{1}$	$N_{2}$	$L_{2}$	$p_{2}$	$N_{3}$	$L_{3}$	$p_{3}$	$p_{\mathrm{fail}}$	$p_{\mathrm{suc}}$
20	40	0.60269	8	28	0.74756	3	23	0.87515	1.25220	98.7478
50	100	0.60501	20	70	0.74993	6	56	0.89753	1.01220	98.9878
100	200	0.60577	40	140	0.75071	10	110	0.91272	0.85776	99.1422
200	400	0.60615	79	279	0.75302	20	220	0.91291	0.84713	99.1529

## Data Availability

The data that support the findings of this study are available from the corresponding authors upon reasonable request.

## References

[1] Baccelli F., Blaszczyszyn B., Muhlethaler P. (2006). An Aloha protocol for multihop mobile wireless networks. IEEE Trans. Inf. Theory.

[2] Borgonovo F., Capone A., Cesana M., Fratta L. (2004). ADHOC MAC: New MAC Architecture for Ad Hoc Networks Providing Efficient and Reliable Point-to-Point and Broadcast Services. Wirel. Netw..

[3] Han C., Dianati M., Tafazolli R., Liu X., Shen X. (2012). A Novel Distributed Asynchronous Multichannel MAC Scheme for Large-Scale Vehicular Ad Hoc Networks. IEEE Trans. Veh. Technol..

[4] Wang Y., Shi J., Chen L., Lu B., Yang Q. (2019). A Novel Capture-Aware TDMA-Based MAC Protocol for Safety Messages Broadcast in Vehicular Ad Hoc Networks. IEEE Access.

[5] Choi S.S., Kim S. (2009). A dynamic framed slotted aloha algorithm using collision factor for RFID identification. IEICE Trans. Commun..

[6] Ferreira H.P.A., Assis F.M.D., Serres A.R. (2019). A Novel RFID Method for Faster Convergence of Tag Estimation on Dynamic Frame Size ALOHA Algorithms. IET Commun..

[7] Cassará P., Cola T.D., Gotta A. (2020). A Statistical Framework for Performance Analysis of Diversity Framed Slotted Aloha With Interference Cancellation. IEEE Trans. Aerosp. Electron. Syst..

[8] Wang Y., Shi J., Chen L. (2018). Capture Effect in the FSA-Based Networks under Rayleigh, Rician and Nakagami-m Fading Channels. Appl. Sci..

[9] Hadzi-Velkov Z., Spasenovski B. Capture effect in IEEE 802.11 basic service area under influence of Rayleigh fading and near/far effect. Proceedings of the 13th IEEE International Symposium on Personal, Indoor and Mobile Radio Communications.

[10] Tian N., Cai X., Cheng J., Yue W., Luo M. (2022). Short-Packet Transmission in Irregular Repetition Slotted ALOHA System over the Rayleigh Fading Channel. Int. J. Pattern Recognit. Artif. Intell..

[11] Pejoski S., Hadzi-Velkov Z. (2020). Slotted ALOHA Wireless Networks with RF Energy Harvesting in Nakagami-m Fading. Ad Hoc Netw..

[12] Yue Z., Yang H.H., Zhang M., Pappas N. (2023). Age of Information Under Frame Slotted ALOHA-Based Status Updating Protocol. IEEE J. Sel. Areas Commun..

[13] Sun M., Guo Y., Zhang D., Jiang M.M. (2021). Anonymous Authentication and Key Agreement Scheme Combining the Group Key for Vehicular Ad Hoc Networks. Complexity.

[14] Qiong W., Shuai S., Ziyang W., Qiang F., Pingyi F., Cui Z. (2023). Towards V2I Age-aware Fairness Access: A DQN Based Intelligent Vehicular Node Training and Test Method. Chin. J. Electron..

[15] Akyıldız T., Ku R., Harder N., Ebrahimi N., Mahdavifar H. ML-Aided Collision Recovery for UHF-RFID Systems. Proceedings of the 2022 IEEE International Conference on RFID (RFID).

[16] Yu J., Zhang P., Chen L., Liu J., An J. (2020). Stabilizing Frame Slotted Aloha Based IoT Systems: A Geometric Ergodicity Perspective. IEEE J. Sel. Areas Commun..

[17] Bacco M., Cassara P., Gotta A., Cola T.D. Diversity Framed Slotted Aloha with Interference Cancellation for Maritime Satellite Communications. Proceedings of the ICC 2019–2019 IEEE International Conference on Communications (ICC).

[18] Rajeswar R.G., Ramanathan R. (2018). An Empirical study on MAC layer in IEEE 802.11p/WAVE based Vehicular Ad hoc Networks. Procedia Comput. Sci..

[19] Liu J.X., Ding S.B., Zhang L., Xie X.P. (2023). Event-driven intermittent control for vehicle platooning over vehicular ad hoc networks. Int. J. Robust Nonlinear Control.

[20] Shah A.F.M.S., Ilhan H., Tureli U. Modeling and Performance Analysis of the IEEE 802.11 MAC for VANETs under Capture Effect. Proceedings of the 2019 IEEE 20th Wireless and Microwave Technology Conference (WAMICON).

[21] Menouar H., Yagoubi M.B., Ouladdjedid L.K. (2018). CSSA MAC: Carrier sense with slotted-Aloha multiple access MAC in vehicular network. Int. J. Veh. Inf. Commun. Syst..

[22] Chowdhury M.S., Ullah N., Al Ameen M., Kwak K.S. (2015). Framed slotted aloha based MAC protocol for low energy critical infrastructure monitoring networks. Int. J. Commun. Syst..

[23] Kim Y., Lee M., Lee T.J. (2016). Coordinated Multichannel MAC Protocol for Vehicular Ad Hoc Networks. IEEE Trans. Veh. Technol..

[24] Qi H.M., Chen J.Q., Yuan Z.Y., Fan L.L. A Low Energy Consumption and Low Delay MAC Protocol Based on Receiver Initiation and Capture Effect in 5G IoT. Proceedings of the Algorithms and Architectures for Parallel Processing: 21st International Conference.

[25] Jeong J., Choi S., Yoo J., Lee S., Kim C.K. (2013). Physical layer capture aware MAC for WLANs. Wirel. Netw..

[26] Sanchez-Garcia J., Smith D.R. (2002). Capture probability in Rician fading channels with power control in the transmitters. Commun. IEEE Trans..

